# Supplementary figures and images for: Metabolic adaptations underlying genome flexibility in prokaryotes
Source: PLoS Genet. 2018 Oct 29;14(10):e1007763. doi: 10.1371/journal.pgen.1007763 (PMC6224172; doi:10.1371/journal.pgen.1007763)

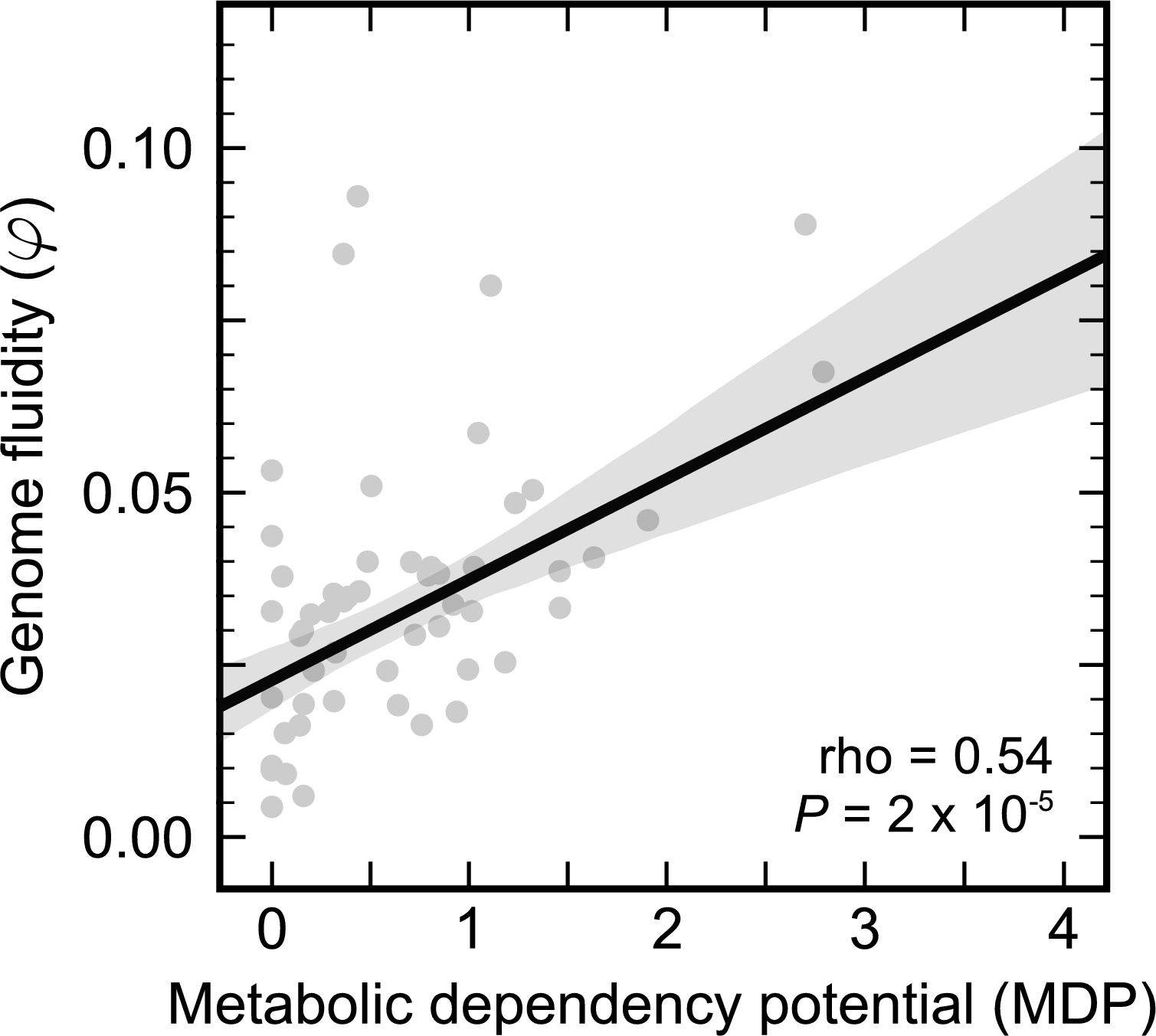

Supplement: S1 Fig — Scatter plot of genome fluidity φ versus conspecific metabolic dependency potential (MDP), similar to Fig 2, but to minimize phylogenetic bias, here I only included one species per genus. This resulted in 55 species (51 bacteria, 4 archaea). Each point represents the average number of dependencies detected per strain per condition across all conspecific pairs for one species. The solid black line represents a linear regression and the gray envelope around it, the 95% prediction interval. MDP still increases significantly with increasing genome fluidity. (TIF) [file pgen.1007763.s001.tif]

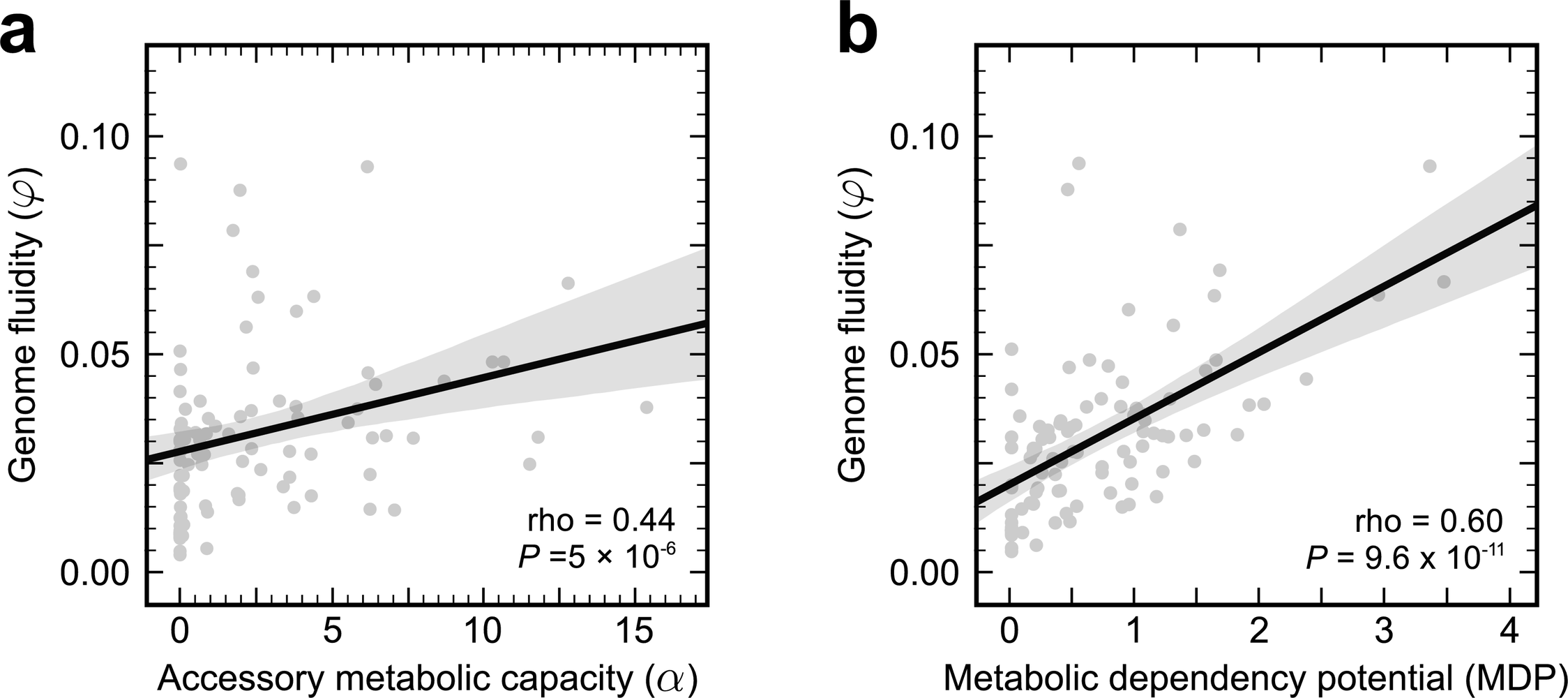

Supplement: S2 Fig — a, Scatter plot of genome fluidity φ versus accessory metabolic capacity α for the 96 prokaryotic species in this study, similar to Fig 1; and b, scatter plot of genome fluidity φ versus conspecific metabolic dependency potential (MDP), similar to Fig 2; except without the addition of any gap-filled reactions (see Methods). This eliminates any potential bias that may arise from adding gap-filled reactions. Each point represents one species, both solid black lines represent linear regression, and the gray envelopes around them, 95% prediction intervals. Both observed trends are qualitatively unaffected. (TIF) [file pgen.1007763.s002.tif]

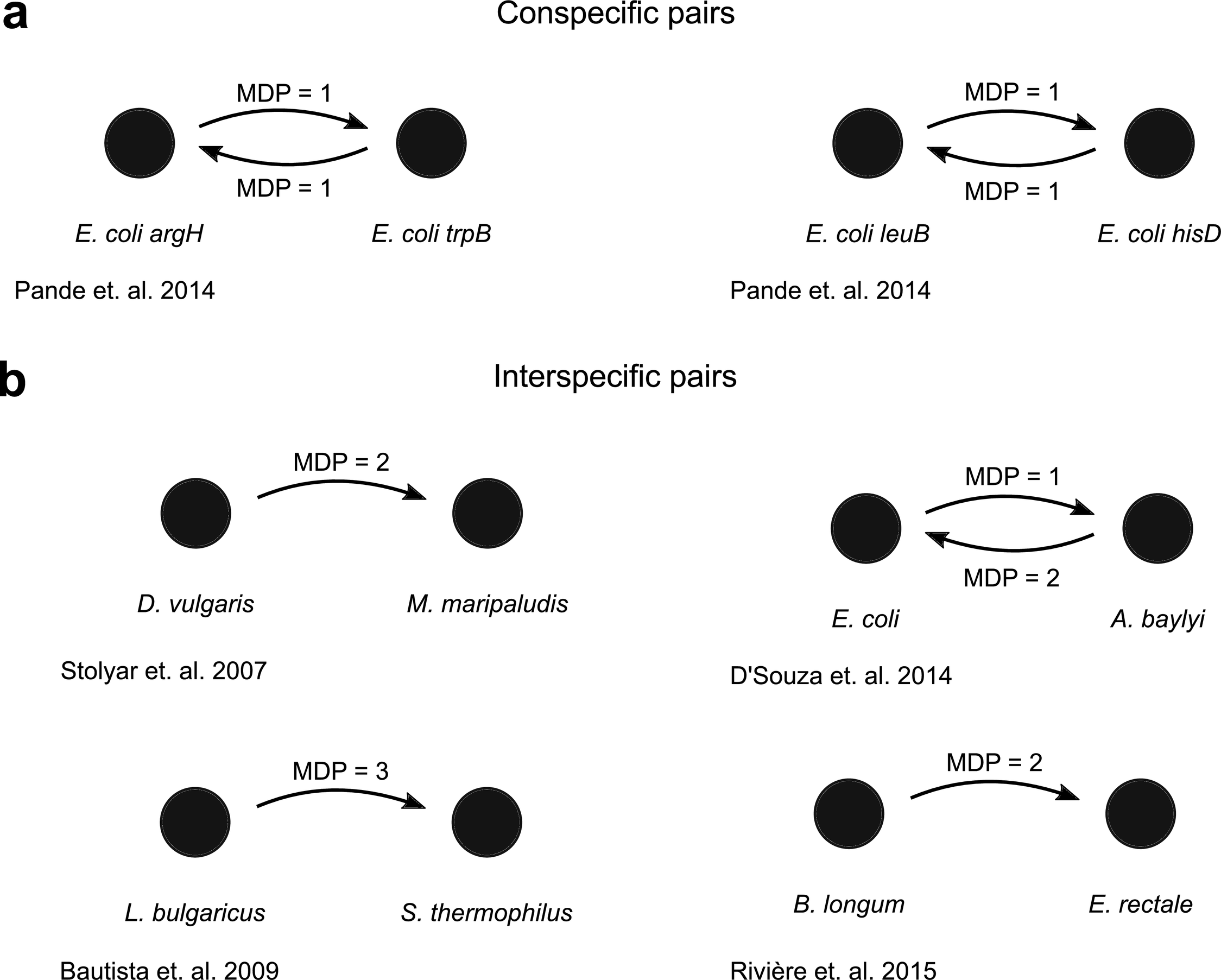

Supplement: S3 Fig — For a, 2 conspecific and b, 4 interspecific pairs of prokaryotes, I verified that my approach to infer and measure metabolic dependencies using KEGG-annotated metabolic reaction networks (see Methods) can predict both the number of dependencies between each microbe, as well as the correct interaction type (commensalism, as in the top-left pair in b; and mutualism, as in the top-right pair). (TIF) [file pgen.1007763.s003.tif]

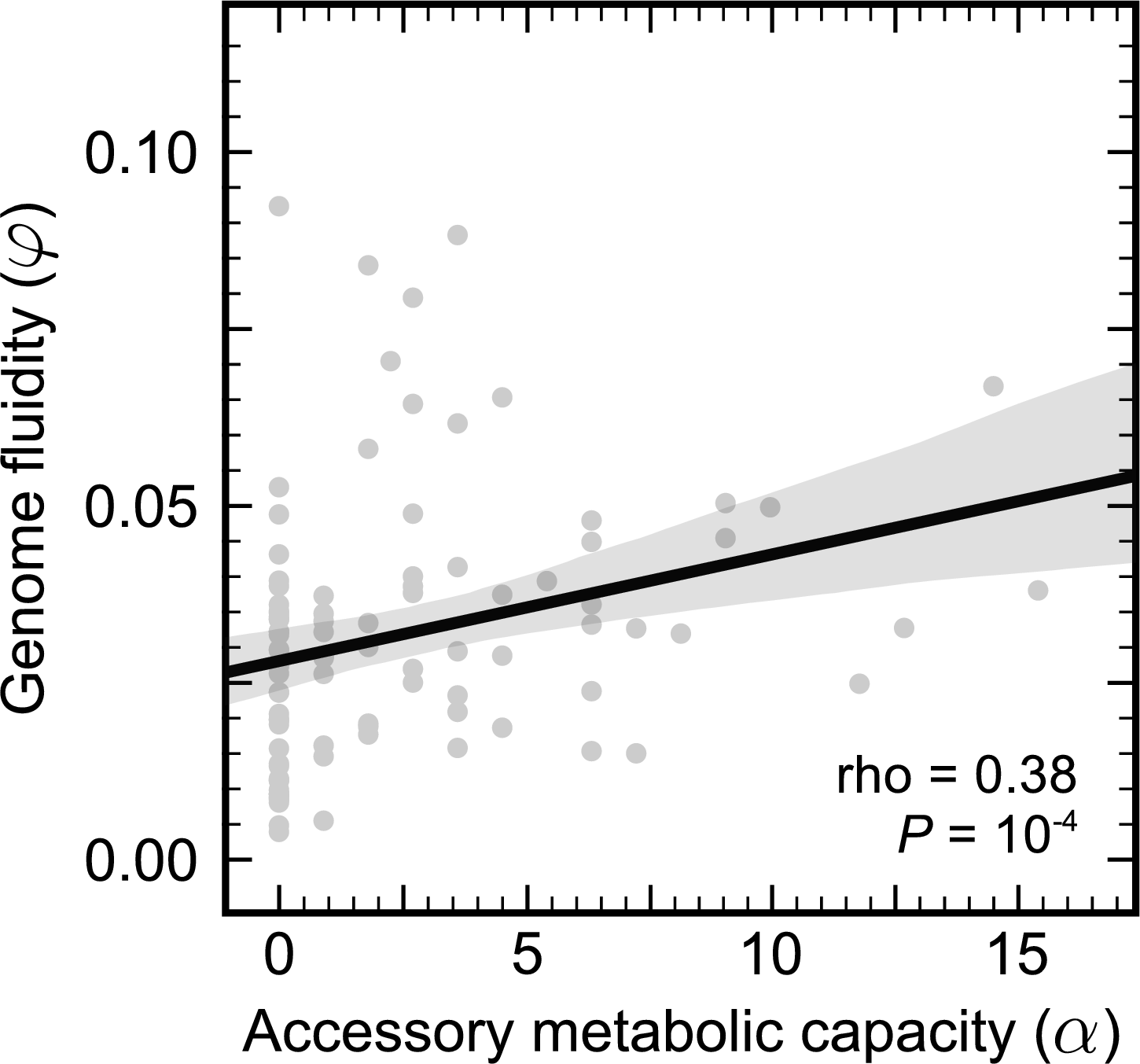

Supplement: S4 Fig — Same as Fig 1, except here to calculate α, instead of using the mean number of precursors produced by each individual strain’s accessory genome, I considered medians. The solid black line represents a linear regression and the gray envelope around it, the 95% prediction interval. rho corresponds to Spearman’s nonparametric correlation coefficient and the P value to a one-way asymptotic permutation test for positive correlation. This choice does not significantly impact my results for α. (TIF) [file pgen.1007763.s004.tif]

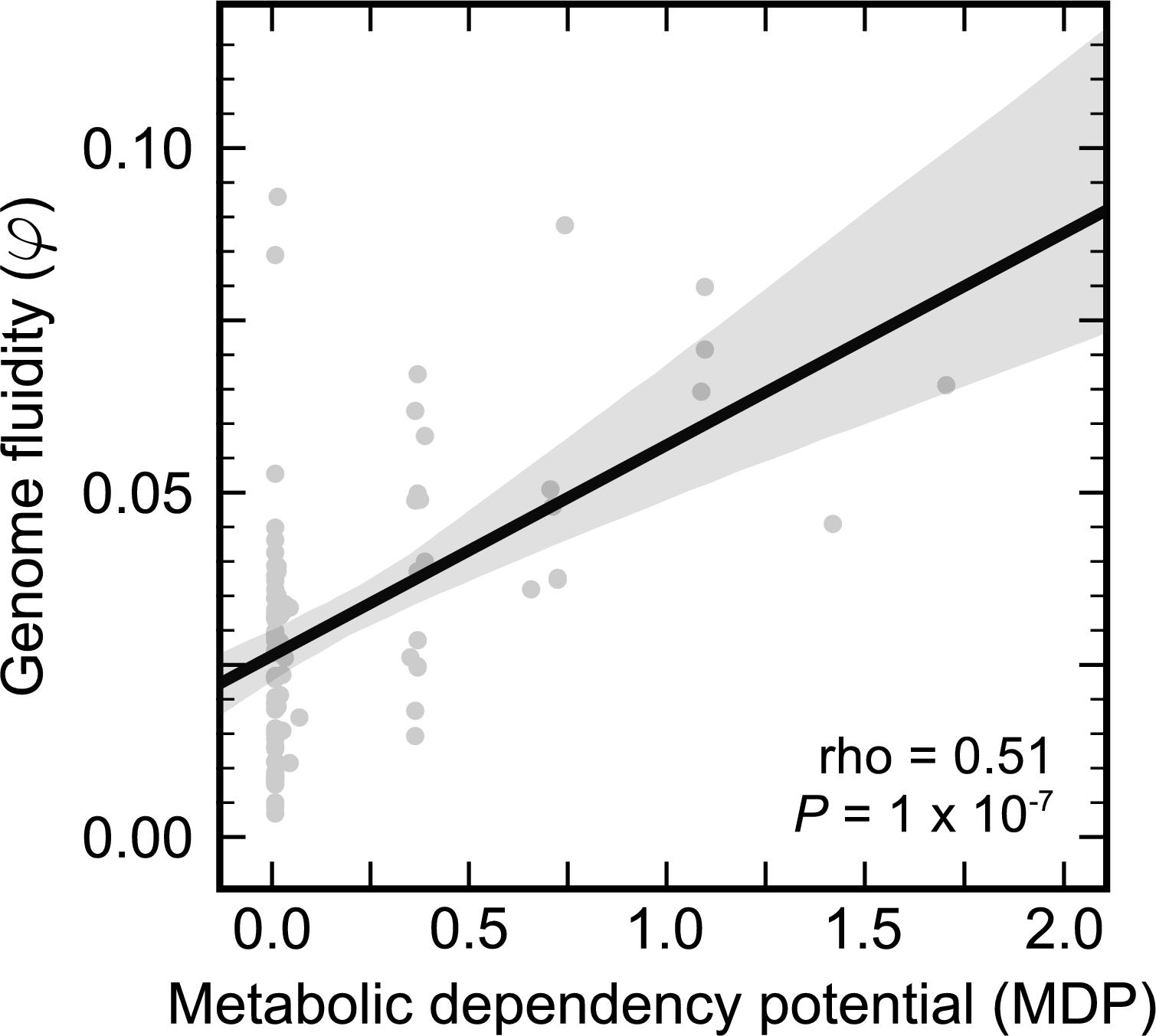

Supplement: S5 Fig — Same as Fig 2a, except here to calculate α, instead of using the mean number of dependencies per strain across conspecific pairs, I considered medians. The solid black line represents a linear regression and the gray envelope around it, the 95% prediction interval. rho corresponds to Spearman’s nonparametric correlation coefficient and the P value to a one-way asymptotic permutation test for positive correlation. This choice does not significantly impact my results for MDP. (TIF) [file pgen.1007763.s005.tif]

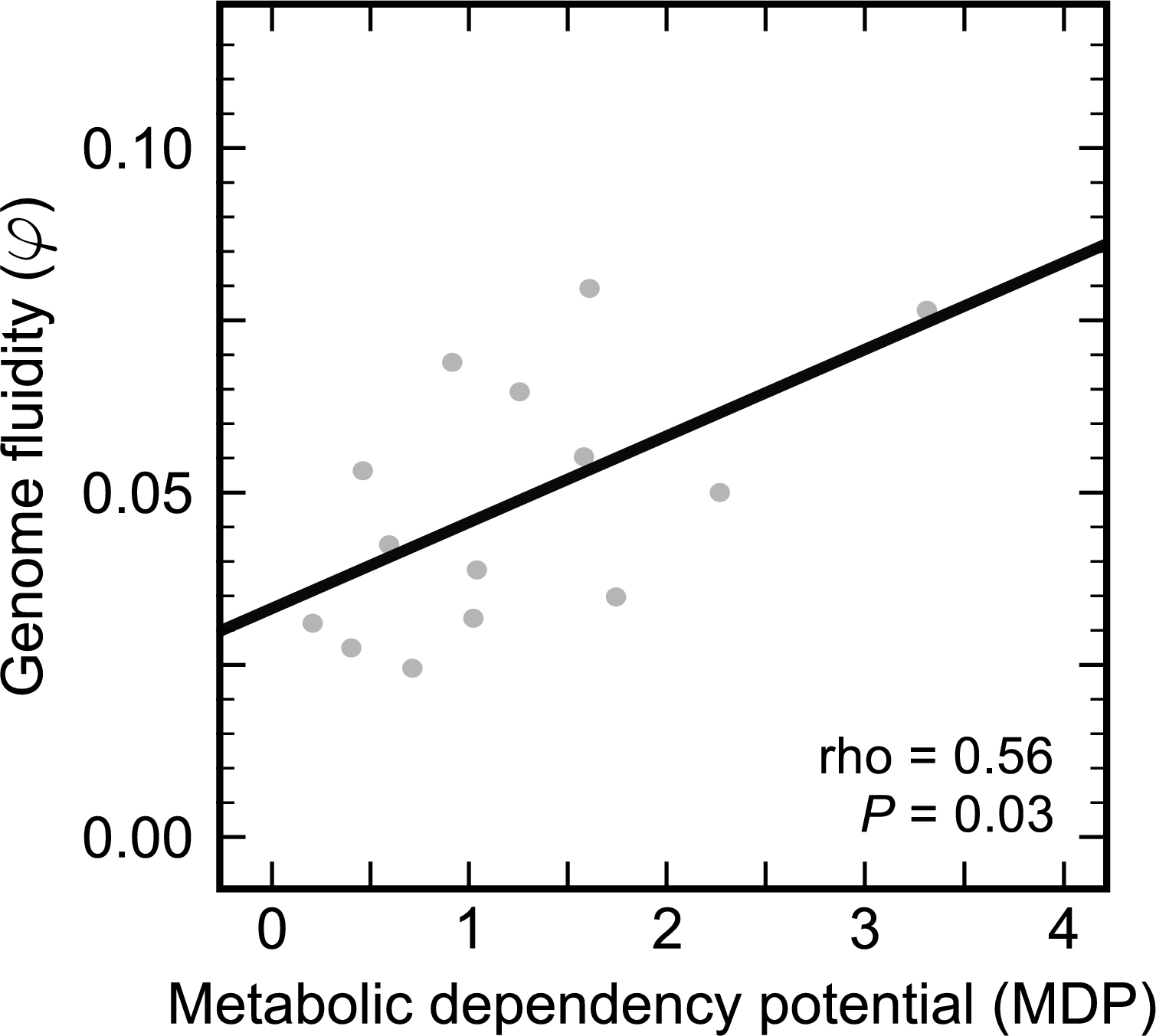

Supplement: S6 Fig — Scatter plot of genome fluidity φ versus conspecific metabolic dependency potential (MDP), similar to Fig 2, but to test if the detected dependencies are realizable in nature, here I only included known co-occurring strains (see Methods). This resulted in 29 strains across 14 species (S7 Table). Each point represents the average number of dependencies detected per strain per condition across all co-occurring conspecific pairs for one species. The solid black line represents a linear regression and the gray envelope around it, the 95% prediction interval. MDP still increases significantly with increasing genome fluidity. (TIF) [file pgen.1007763.s006.tif]

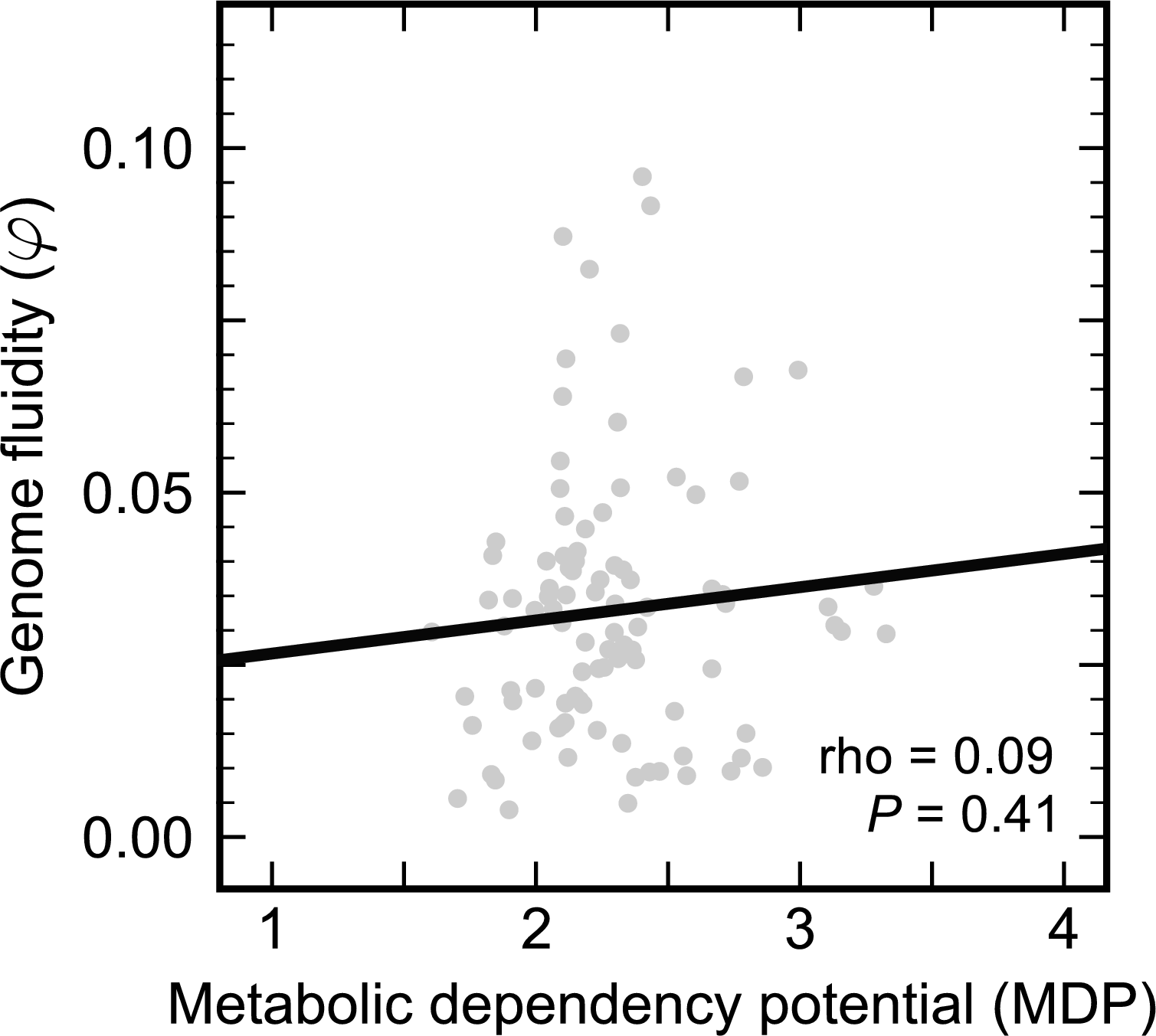

Supplement: S7 Fig — Scatter plot of genome fluidity φ versus conspecific metabolic dependency potential (MDP), similar to Fig 2, but here MDP was measured between inter-specific strains (see Methods). For each strain within a species, I measured its dependency potential with 25 randomly chosen strains from other species. Each point represents the average number of dependencies detected per strain per condition across several inter-specific pairs for one species. The solid black line represents a linear regression and the gray envelope around it, the 95% prediction interval. MDP is nonzero for all species in the study, though it does not increase significantly with increasing genome fluidity. (TIF) [file pgen.1007763.s007.tif]

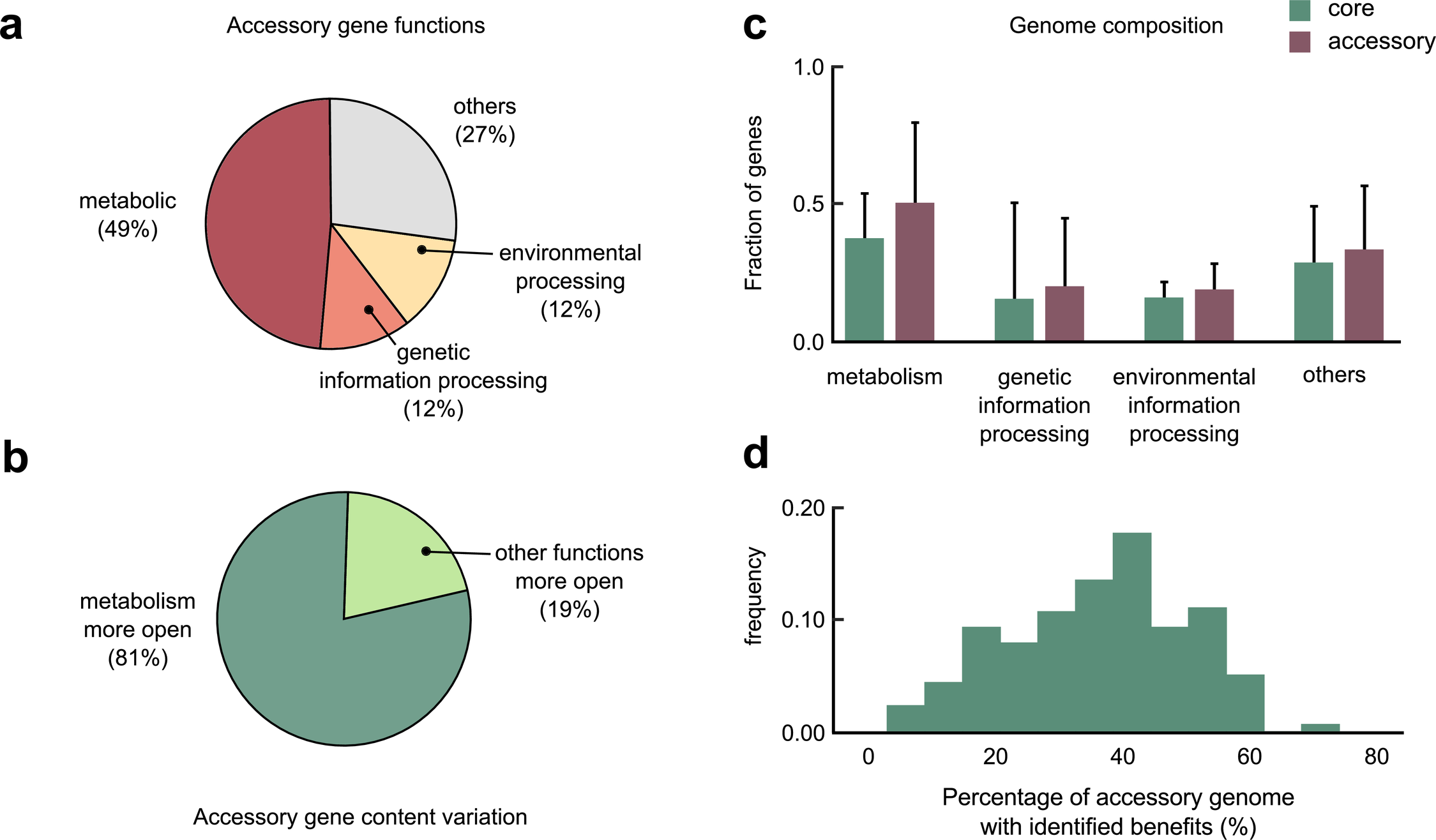

Supplement: S8 Fig — a, Pie chart of the average fraction of genes belonging to different functions typical to prokaryotic accessory genomes (for the 1,339 genomes in this study). b, Pie chart of the fraction of 96 species in the study where metabolic functions showed more gene content variation than other functions in accessory genomes (namely, genetic information processing, environmental information processing, and others). c, Bar chart of the typical functional composition of both core and accessory genomes, for the 96 species in the study. Values indicate median fraction, and error bars indicate the extent of variation observed across the genomes studied. Metabolic functions are enriched in accessory genomes when compared with core genomes. d, Histogram of the fraction of accessory genes in each strain, which I identified as potential contributors to metabolically beneficial functions in the study. (TIF) [file pgen.1007763.s008.tif]

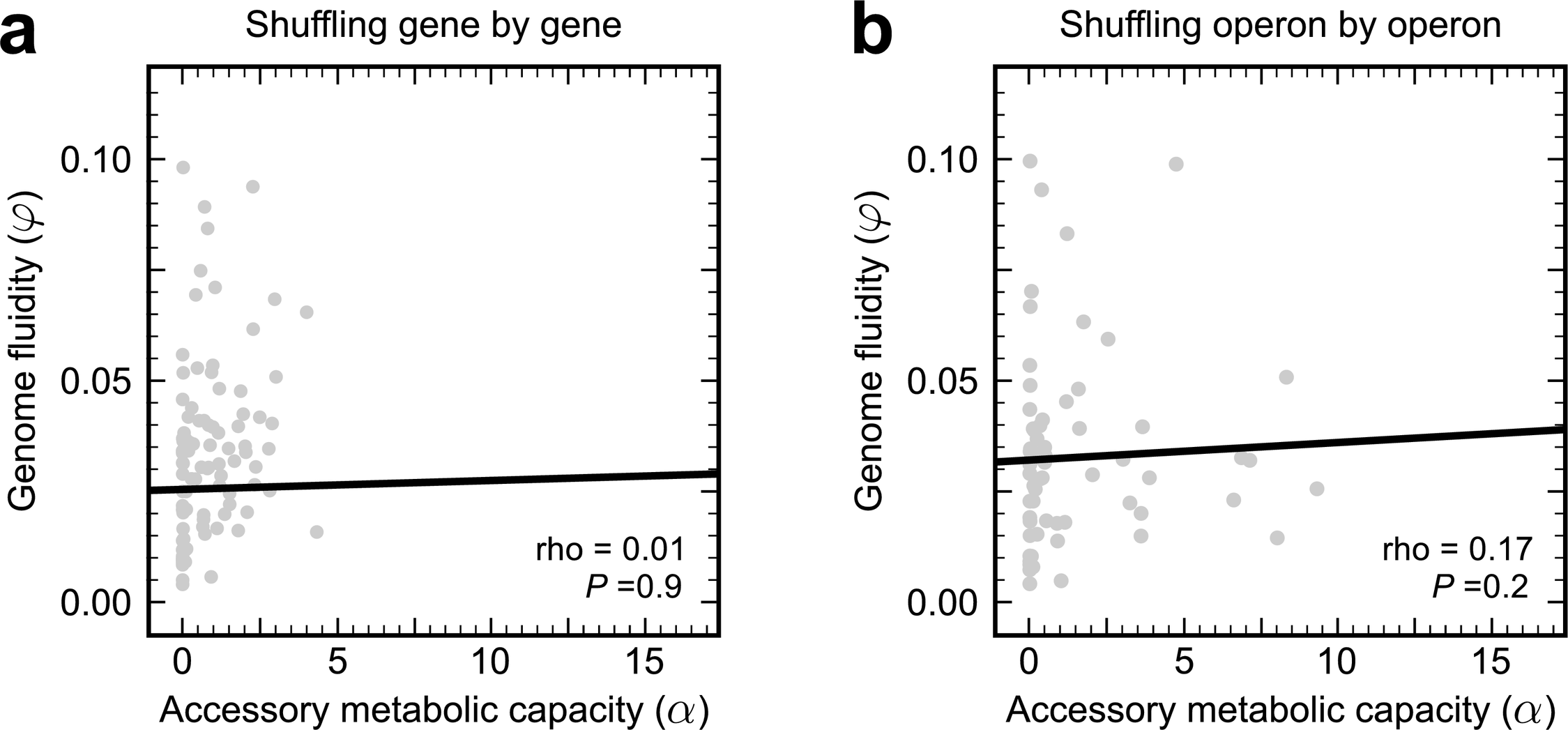

Supplement: S9 Fig — Scatter plot of genome fluidity α versus accessory metabolic capacity α for the prokaryotic species in this study, with each species’ accessory content randomly shuffled between strains, either a, gene by gene (for 96 species), or b, operon by operon (for 64 species, see Methods). The solid black lines represent linear regression. In both cases, not only does randomly shuffling accessory genes significantly reduce the additional biosynthetic potential of each strain’s accessory genome, there is no significant correlation with increasing accessory content (compared with Fig 1). (TIF) [file pgen.1007763.s009.tif]

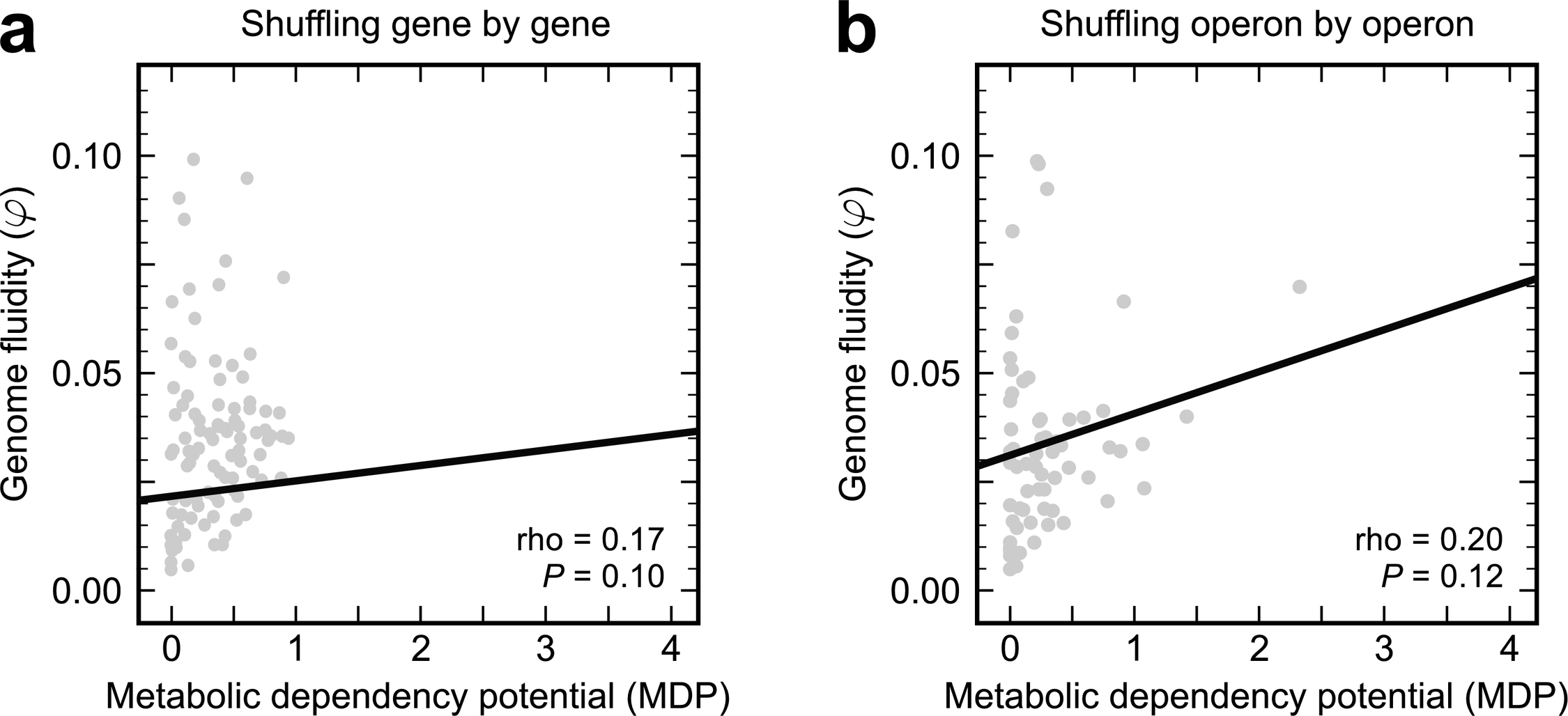

Supplement: S10 Fig — Scatter plot of genome fluidity φ versus metabolic dependency potential MDP for the prokaryotic species in this study, with each species’ accessory content randomly shuffled between strains, either a, gene by gene (for 96 species), or b, operon by operon (for 64 species, see Methods). The solid black lines represent linear regression. In both cases, not only does randomly shuffling accessory genes significantly reduce the number of metabolic dependencies between conspecific pairs, there is no significant correlation with increasing accessory content (compared with Fig 2a). (TIF) [file pgen.1007763.s010.tif]

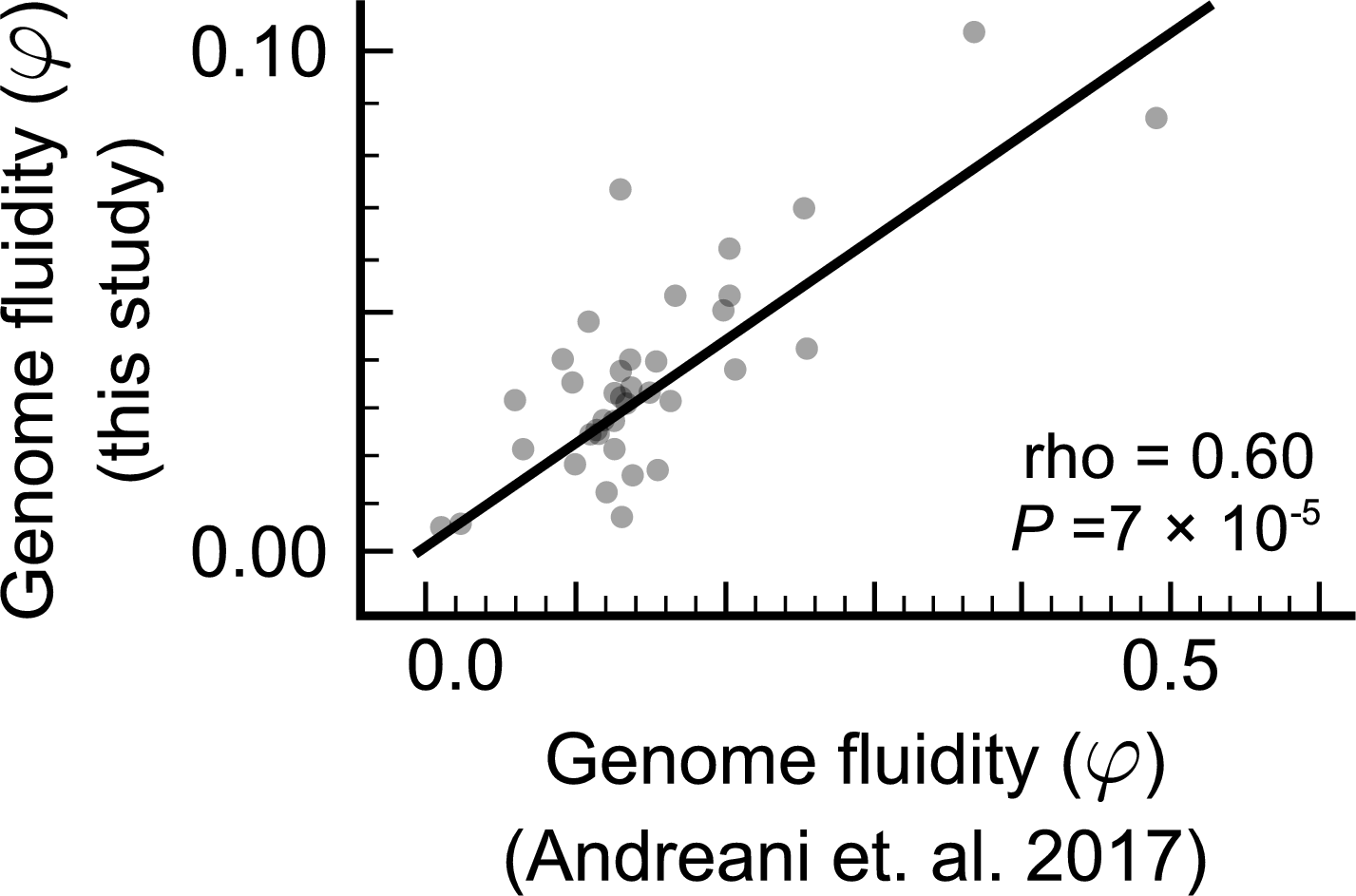

Supplement: S11 Fig — Scatter plot of genome fluidity φ using KEGG (this study; y-axis) versus estimates of φ reported in Andreani et. al., The ISME Journal (2017) (x-axis) for the 38 species common to both analyses. Each point represents one species. My measure uses KEGG’s orthologous gene categories for scoring gene presence-absence, whereas Andreani et. al. use thresholded sequence alignment to classify genes into “families”. The solid black line represents a linear regression. rho corresponds to Spearman’s nonparametric correlation coefficient and P to a one-way asymptotic permutation test for positive correlation. While my estimates are lower than those of Andreani et. al., they show consistent linear scaling and can thus still be used to infer trends. (TIF) [file pgen.1007763.s011.tif]
